# Therapeutic aspects of cell signaling and communication in Duchenne muscular dystrophy

**DOI:** 10.1007/s00018-021-03821-x

**Published:** 2021-04-07

**Authors:** Alicja Starosta, Patryk Konieczny

**Affiliations:** Faculty of Biology, Institute of Human Biology and Evolution, Adam Mickiewicz University, ul. Uniwersytetu Poznańskiego 6, 61-614 Poznań, Poland

**Keywords:** Duchenne muscular dystrophy, DMD, Signaling pathways, Striated muscles

## Abstract

Duchenne muscular dystrophy (DMD) is a devastating chromosome X-linked disease that manifests predominantly in progressive skeletal muscle wasting and dysfunctions in the heart and diaphragm. Approximately 1/5000 boys and 1/50,000,000 girls suffer from DMD, and to date, the disease is incurable and leads to premature death. This phenotypic severity is due to mutations in the *DMD* gene, which result in the absence of functional dystrophin protein. Initially, dystrophin was thought to be a force transducer; however, it is now considered an essential component of the dystrophin-associated protein complex (DAPC), viewed as a multicomponent mechanical scaffold and a signal transduction hub. Modulating signal pathway activation or gene expression through epigenetic modifications has emerged at the forefront of therapeutic approaches as either an adjunct or stand-alone strategy. In this review, we propose a broader perspective by considering DMD to be a disease that affects myofibers and muscle stem (satellite) cells, as well as a disorder in which abrogated communication between different cell types occurs. We believe that by taking this systemic view, we can achieve safe and holistic treatments that can restore correct signal transmission and gene expression in diseased DMD tissues.

## Introduction

Duchenne muscular dystrophy (DMD) is a chromosome X-linked disease that affects approximately 1/5000 boys and 1/50,000,000 girls [1–3]. DMD manifests in progressive skeletal muscle wasting and heart and diaphragm dysfunctions [4]. Depending on the dystrophin expression profile, some patients also suffer from other phenotypic alterations that include cognitive deficits and psychiatric problems [5, 6]. DMD is typically diagnosed around the age of 5 when the first symptoms of motor delay or abnormal gait become apparent, although preceding delays in independent walking and language development may have also been observed [7]. With progression of the disease, the extent of muscle fiber loss is so vast that patients are forced to use a wheelchair and respiratory system aid to help the diaphragm sustain breathing. To date, DMD is incurable and leads to premature death by the twenties or thirties due to respiratory or cardiac failure [7].

DMD is caused by mutations in the *DMD* gene that lead to the absence of functional dystrophin protein. A number of research studies have revealed that a lack of functional dystrophin results in aberrant signal transduction, which leads to epigenome modifications and altered gene expression in affected muscle tissues [8–11]. Although still limited, knowledge regarding dystrophin absence-associated disturbed signaling resulted in the first experimental therapies and clinical trials based on gene expression modulation. For example, the use of histone deacetylase inhibitors showed promising therapeutic outcomes in DMD animal models and patients [12, 13]. Nonetheless, due to the systemic and nonspecific action of these therapies, disadvantageous side effects are inevitable [12]. We are only starting to recognize the intricate web of interactions between myofiber and its environment, which consists of various cell types, including muscle stem (satellite) cells, fibro/adipogenic progenitors (FAPs), immune cells, motoneurons, bone cells, and blood vessel cells. Any of the therapeutic approaches based on tweaking gene expression in the diseased tissue would need to take into account the magnitude of these cell-to-cell interactions. In this article, we gather and review the available data on the interdependence of various cell types in the muscle and surrounding tissues in the context of dystrophin deficiency.

## Molecular background of Duchenne muscular dystrophy

*DMD* is localized on the Xp21 locus, which spans ~ 2.5 Mbp and consists of 79 exons. Its activation leads to the synthesis of several mRNA transcripts in a tissue-dependent manner. In particular, a 14 kb mRNA encoding 427 kDa dystrophin isoform (Dp427) is generated in high amounts in skeletal muscles and the heart [14], and all mutations causing DMD result in either the loss or production of highly dysfunctional Dp427 [15]. Dp427 synthesis occurs at three distinct promoters. Dp427m is present in skeletal and cardiac muscles, Dp427c occurs in neurons of the cortex and the CA regions of the hippocampus, and Dp427p is found in cerebellar Purkinje cells [14]. In addition to full-length dystrophin, several shorter isoforms are generated as a result of transcription from several other unique promoters. Isoforms Dp260 and Dp116 are observed mainly in the retina and peripheral nerves, respectively [16, 17], while Dp140 is predominantly synthesized during fetal stages and in the adult brain, similar to the shortest Dp40 [18, 19]. In contrast, Dp71 is ubiquitously present in various tissues, and although previously considered to be a nonmuscle isoform [20, 21], its presence has been recently confirmed in myoblasts and myofibers [21, 22]. Depending on the site of mutation in *DMD*, the expression of shorter dystrophins might also be compromised [23], which impacts the extent of the observed pathological alterations. For instance, mutations that affect the expression of Dp71, which is especially abundant in the brain, account for intellectual disability in DMD patients [5].

**Fig. 1:**
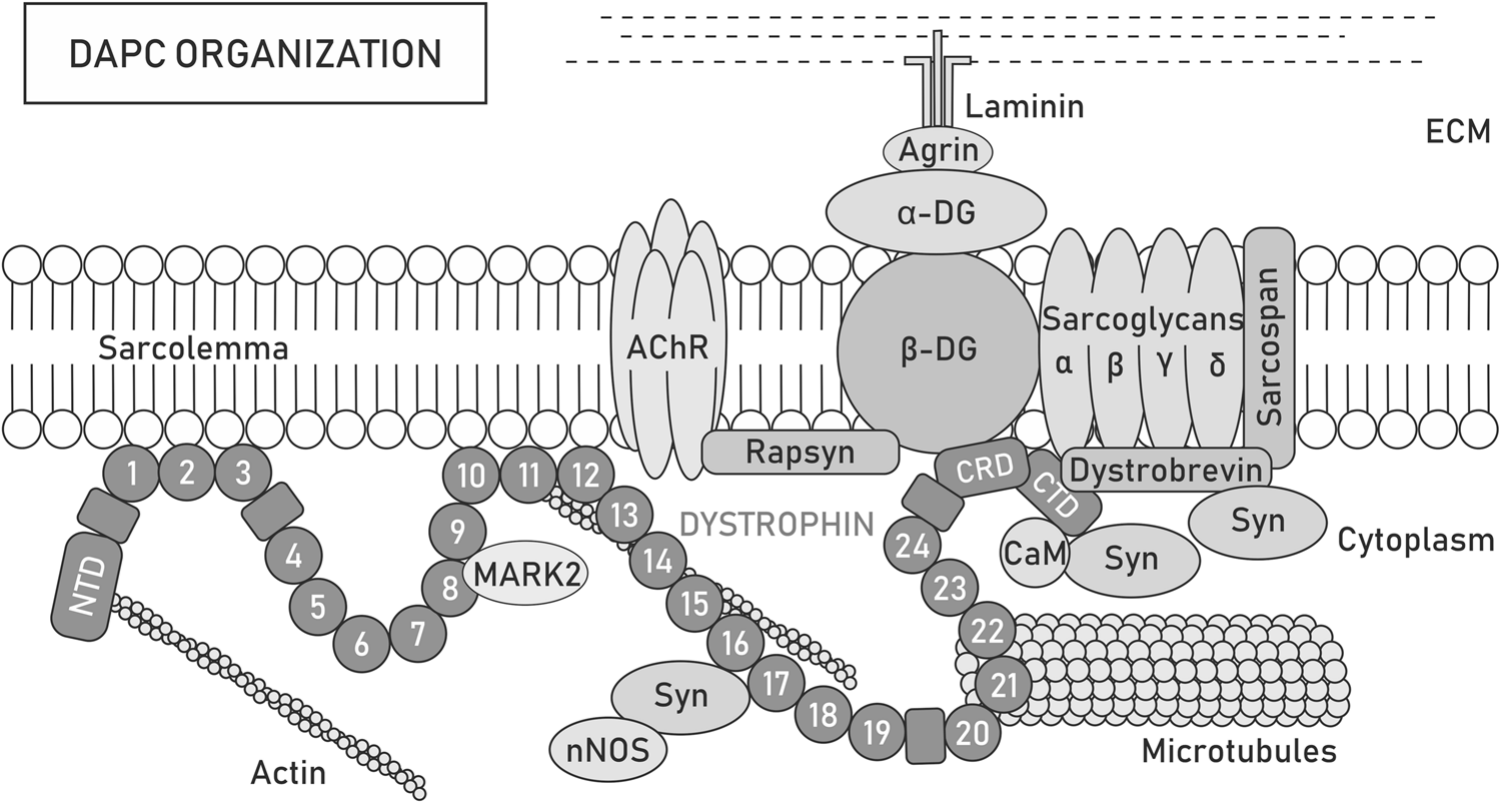
Dystrophin-associated protein complex organization. Dystrophin forms a scaffold for the dystrophin-associated protein complex (DAPC) that acts both as a mechanical force transducer and a signaling hub. Depending on the presence of the dystrophin isoform, its paralog utrophin or tissue/cellular localization, the content of DAPC may differ. Full-length dystrophin Dp427 consists of the N-terminal domain (NTD), central rod [with 24 spectrin-like repeats (circles) and 4 hinge modules (rectangles)], cysteine-rich (CRD), and C-terminal (CTD) domains, which provide multiple sites for interactions with proteins. NTD and spectrin-like repeats 11–15 bind costameric actin filaments, while repeats 8 and 9 anchor MARK2 and 20–23 interact with microtubules, which can also contact dystrophin indirectly through ankyrin-B. Spectrin-like repeats 1–3 and 10–12 may additionally participate in stabilizing the complex to the sarcolemma by binding the lipid bilayer. Through CRD, dystrophin interacts with sarcolemma-located β-dystroglycan (β-DG), which is anchored to α-dystroglycan (α-DG). In turn, α-DG binds laminin, a component of the extracellular matrix (ECM) network, which facilitates the transfer of forces during muscle contraction from the cytoskeleton to the ECM and protects the sarcolemma from twich-induced damage. At the neuromuscular junction (NMJ), the interaction of α-DG with agrin and perlecan provides MuSK-induced clustering of acetylcholine receptors (AChRs) and determines the localization of acetylcholine esterase, while the binding of β-DG with rapsyn is involved in the clustering of AChRs. Additionally, β-DG stabilizes α, β, δ- and γ-sarcoglycan and the sarcospan complex at the sarcolemma. The CTD of dystrophin interacts with various cytosolic proteins, such as dystrobrevin or syntrophins (Syn). Syntrophins recruit sodium channels and signaling molecules, such as neuronal nitric oxide synthase (nNOS)

Initially, dystrophin was considered to serve only mechanical functions, particularly in the context of the dystrophin-associated protein complex (DAPC) assembled in the sarcolemma and known to transduce the force during muscle contraction to the extracellular matrix (ECM) [24]. Over time, interactions of either dystrophin or DAPC with several cytoskeletal elements and cell signaling molecules have been reported [25]. Because of its numerous binding domains and interaction partners, dystrophin is now considered part of a multicomponent mechanical scaffold as well as a signal transduction hub [26–29] (Fig. 1).

The lack of functional full-length dystrophin (Dp427) inevitably leads to sarcolemma destabilization and muscle degradation. These effects are particularly evident in myofibers that are stretched during contractions, where dystrophin absence results in extensive membrane tearing, influx of Ca^2+^ through stretch-activated ion channels, a rise in intracellular Ca^2+^ levels and the associated overproduction of reactive oxygen species (ROS) [30, 31]. This process is considered an important initiating event in dystrophic pathogenesis and a subject of many studies that focus on therapeutic interventions in DMD [32–35]. The absence of dystrophin and the resultant disorganization of DAPC also impair the distribution of proteins at and underneath the sarcolemma, including signaling molecules, additionally contributing to myofiber death [36]. Importantly, dystrophin deficiency seems to be naturally alleviated by expression of the utrophin (*UTRN*) gene in both humans and animal models [37, 38], which is signified by the structural and functional similarities between utrophin and dystrophin [39, 40] and by the differences in the severity of the phenotype of various DMD mouse models. In particular, *mdx* mice that lack Dp427 have relatively mild disease symptoms and only slightly affected lifespans [41, 42], while mice missing both dystrophin and utrophin (*mdx/utrn*^*−/−*^) show a much more severe phenotype and usually die before the age of 20 weeks [43, 44]. Despite clear symptomatic differences in the course and severity of the disease in *mdx* mice and humans, *mdx* mice continue to be the most commonly used animal model of DMD [41, 45]. The effect of an absence of dystrophin in the muscle is not limited to the myofiber pathology but also pertains to the direct loss of dystrophin-related functions in satellite cells and vasculature [46, 47]. In particular, loss of dystrophin in satellite cells results in alterations in their division kinetics and differentiation into mature fibers [46].

Treatment strategies for DMD target primary defects or attenuate secondary downstream pathologies. The first approach aims to restore functional dystrophin protein. For example, antisense oligonucleotide-mediated exon skipping targets pre-mRNA splicing to restore shortened but functional proteins [48], while the CRISPR/Cas9 system is used to edit the defective *DMD* gene [49]. Currently, four exon skipping antisense oligonucleotides are FDA-approved for use in the treatment of DMD patients: Amondys 45 (casimersen), Viltepso (viltolarsen), Exondys 51 (eteplirsen) and Vyondys 53 (golodirsen)  [50–52]. Moreover, CRISPR/Cas9-based strategies have provided promising therapeutic approaches; however, limitations remain, including the risk of off-target gene editing, and further research is required [50]. The above strategies are mutation-specific and thus are not universal and cannot be used for all patients. In contrast, the sequence of functional dystrophin can be delivered to all cells with recombinant adeno-associated viral (rAAV) vectors, although their capacity is limited to approximately 4.7 kb and thus precludes insertion of a cassette spanning the 11 kb coding fragment of Dp427 mRNA. Importantly, vectors carrying microdystrophins to muscle fibers are in clinical trials with promising initial results [53, 54] and minidystrophins can be generated by dual rAAV expression vectors [55, 56]. Cell-based therapies offer another approach; however, despite promising studies in animal models, the results are not fully transferable to humans, and the limitations include poor survival and limited migration (myoblasts), quick differentiation that limits their regenerative potential (satellite cells; SCs), risk of thalamic stroke (mesangioblasts), and limited ability to differentiate into muscle cells (bone marrow cells and CD133 + cells) [37, 57].

All these therapeutic methods must face the problem of immune rejection of either the restored or delivered dystrophin, as well as other foreign proteins, including viral capsids. Alternative strategies that bypass the problem of potential immune rejection consist of (1) genetically corrected autologous pluripotent stem cells differentiated ex vivo into dedicated muscle stem cells [58, 59] and (2) therapies grounded on either pharmacological induction of the *UTRN* gene [57] or delivery of vectors encoding micro- and mini-utrophins [40, 60]. Importantly, the Davies and Chamberlain groups revealed the therapeutic potential of full-length [61] and truncated utrophins in the muscles of dystrophic mice [62, 63]. Moreover, increasing the level of utrophin in DMD patients using pharmacological activators also had positive outcomes [40]. Nevertheless, it is important to note that the synthesis of dystrophin and utrophin from several promoters, extensive alternative splicing of the resultant mRNAs and the presence of alternative polyadenylation sites result in the expression of a whole range of specialized protein products in various cell types in a time- and often space-restricted manner [20]. Thus, inadequate dystrophin isoform matching could lead to unwanted side effects and long-term therapy designed for myofibers with one particular protein may expose latent dystrophin-related phenotypes in tissues other than muscle. The current results indicate that utrophin can partially compensate for the lack of functional Dp427, both in mechanical and signaling activities [64]. However, forced body-wide expression of specific utrophins might lead to unexpected pathologies due to, e.g., a lack of neuronal nitric oxide synthase (nNOS)-related signaling [65] or inadequate organization of microtubules [66].

In this review, we focus on DMD pathogenesis from a broader perspective that includes affected myofibers and satellite cells as well as abrogated cell communication and signaling between various cell types in the muscle and surrounding tissues, including inflammatory cells, bones, microvasculature, and innervation; however, signaling-related topics, such as noncoding RNAs or exosomes, are omitted because they have been previously reviewed in-depth [67–69]. The involvement of dystrophins in cell signaling and communication opens new therapeutic avenues as either adjuvants or stand-alone approaches. In addition, because all DMD patients suffer from skeletal muscle weakness as well as diaphragm and heart failure, signaling-based approaches could be a universal method of treatment and contribute to improving their quality of life.

## Disrupted signaling in skeletal muscle

**Fig. 2:**
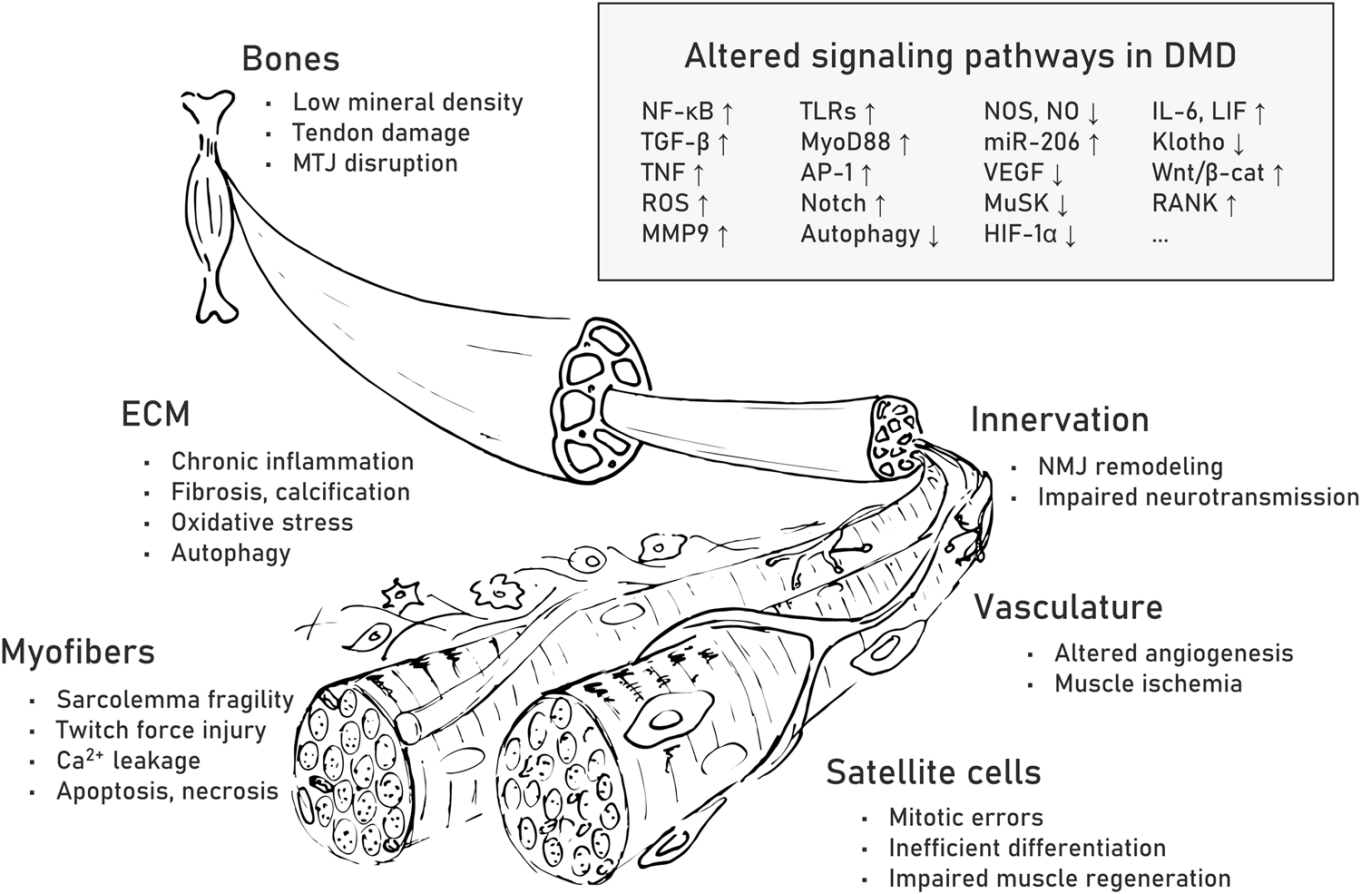
Pathological alterations and affected signaling pathways in DMD muscle fibers and surrounding tissues. Pathological alterations are listed for myofibers as well as extracellular matrix (ECM), bones, innervation, vasculature and satellite cells. Examples of upregulated (↑) and downregulated (↓) signaling pathways associated with the observed pathological changes in muscle fibers and surrounding tissues are listed in the box. Abbreviations: nuclear factor-κB (NF-κB), transforming growth factor-β (TGF-β), tissue necrosis factor (TNF), reactive oxygen species (ROS), matrix metalloprotease 9 (MMP9), Toll-like receptors (TLRs), myeloid differentiation primary response 88 (MyoD88), activator protein 1 (AP-1), neuronal nitric oxide synthase (nNOS), nitric oxide (NO), microRNA-206 (miR-206), vascular endothelial growth factor (VEGF), muscle-specific kinase (MuSK), hypoxia-inducible factor-1α (HIF-1α), interleukin 6 (IL-6), leukemia inhibitory factor (LIF), Wnt/β-catenin (Wnt/β-cat), receptor activator of NF-κB (RANK)

Muscle repair is a highly organized sequence of cellular events resulting in reconstruction of damaged tissue [70]. In DMD, this process is abrogated and shows chronically overlapping degeneration and regeneration cycles caused by DAPC loss and the resultant structural and signaling abnormalities in myofibers and satellite cells. On the one hand, the fibers cannot sustain the contraction forces and die, while on the other hand, they cannot be efficiently regenerated due to the affected division and self-renewal of muscle progenitor cells. Recurring inflammation and progressive fibrosis additionally lead to breakdown of the ECM and further change intracellular signaling as well as compromise interactions between different cell types, including myofibers as well as myoblasts and satellite cells, motoneurons, various bone [71] and microvasculature [72] cells (Fig. 2) and recently discovered FAPs [73].

### Chronic inflammation

Inflammation is a complex biological response in organisms to mitigate harmful stimuli, such as pathogens, irritants, or damaged cells, and initiate tissue repair. It is driven by innate immune response factors (nonspecific, associated with inflammation) and adaptive immune response factors (specialized). The innate immune response is initiated first, and it involves the action of cells, such as neutrophils, monocytes, and macrophages, and soluble factors, including cytokines and complement [74]. The pattern recognition receptors (PRRs) on the membranes of cells of the innate immune response recognize molecules on the pathogen surface as well as released material during cell damage or death, which represent damage-associated molecular patterns (DAMPs). PRRs can be subgrouped depending on the ligand specificity, function, or localization. In particular, Toll-like receptors (TLRs) play a key role in the immune response (Table 1).

**Table 1:** Disrupted signaling pathways in DMD muscles and surrounding tissues

	Signaling pathway	Up- (↑) or downregulated (↓) in DMD	Short description	References for further reading
Muscle inflammation	TLRs	↑	Toll-like receptors (TLRs) belong to the pattern recognition receptors and play crucial roles in the innate immune response. After interaction with damage-associated molecules patterns (DAMPs) or pathogen-associated molecular patterns (PAMPs), TLRs initiate downstream signaling to activate NF-κB, interferons, or mitogen-activated protein kinases (MAPKs) that regulate the expression of immune defense-related genes	[75, 244]
	MyD88	↑	Myeloid differentiation primary response 88 (MyD88) is an adaptor protein for inflammatory signaling pathways, downstream of TLRs and IL-1 receptors. MyD88 links TLRs or IL-1 receptors to IL-1R-associated kinase (IRAK), while activation of IRAK activates NF-κB, MAPKs, and activator protein 1, driving immune response	[245]
	NF-κB	↑	Nuclear factor kappa-light-chain-enhancer of activated B cells (NF-κB) is a family of inducible transcription factors regulating multiple aspects of immune response and inflammation. NF-κB promotes the expression of proinflammatory genes and regulates the survival, activation, and differentiation of immune cells	[246, 247]
	AP-1	↑	Activator protein 1 (AP-1) is a heterodimer transcription factor built of proteins that belong to the Fos, Jun, ATF, and MAF families. In response to growth factor and cytokine signaling, AP-1 controls a wide range of cellular processes, including cell proliferation, death, survival, and differentiation	[248, 271]
	MMPs	↑	Matrix metalloproteinases (MMPs) are zinc-dependent endopeptidases involved in extracellular matrix remodeling, both during physiological processes and in pathological conditions. MMPs are also important players during inflammation	[249]
	TNF	↑	Tumor necrosis factor (TNF) is a pro-inflammatory cytokine that regulates a number of signaling pathways with various downstream effects. TNF proteins are predominantly expressed by immune cells. TNF signaling impacts immune response, inflammation, cell proliferation, programmed cell death, and necrosis	[250]
	Klotho	↓	Klotho proteins are obligate components of endocrine fibroblast growth factor (FGF) receptor complexes and provide the high-affinity binding of FGF19, FGF21, and FGF23 to their receptors. Klotho proteins are known to play a role in aging-related diseases, diabetes, cancer, arteriosclerosis, renal and bone disease, and inflammation processes	[251]
	TGF-β	↑	Transforming growth factor-β (TGF-β) initiates signaling through the canonical SMAD pathway, regulating the expression of hundreds of genes. TGF-β induces also various noncanonical pathways that are responsible for cytoskeleton organization, cell polarity, and miRNA maturation. The effects of TGF-β signaling depend on the cellular context	[252]
	IL	↑	Interleukins (ILs) are a group of cytokines with immunomodulatory functions that play an important role in immune cell differentiation and activation. ILs could have pro- and anti-inflammatory effects, depending on the producing and responding cell type or the phase of the immune response	[253]
Muscle – bone	Wnt/β-cat	↑	Wnt/β-catenin (Wnt/β-cat) pathway regulates cell fate determination, cell migration, polarity and organogenesis during embryogenesis. Binding of Wnt to its membrane receptor causes translocation of β-cat degradation complex to the cell membrane, effecting in accumulation of β-cat in the cytoplasm and its eventual translocation into the nucleus to act as a transcriptional coactivator	[272]
	OPN	↑	Osteopontin (OPN) is a multifunctional protein involved in physiological processes and the pathogenesis of various diseases (e.g., atherosclerosis, cancer, chronic inflammatory diseases). OPN interacts with several integrins and therefore controls cell migration, adhesion, and survival. Additionally, OPN promotes inflammation and regulates biomineralization	[254]
	RANK/RANKL/OPG	↑	Receptor activator of NF-κB (RANK), its ligand (RANKL), and osteoprotegerin (OPG) form the triad of the ligand/signaling receptor/decoy receptor. RANKL, RANK, and OPG have pivotal roles in the regulation of bone metabolism and the immune system. The triad is involved in diverse physiological and pathological contexts, including muscle metabolism	[255]
	IL-6	↑	Interleukin 6 (IL-6) can act both as a pro-inflammatory cytokine and an anti-inflammatory myokine, depending on the cellular context. Additionally, IL-6 stimulates osteoclastogenesis	[78, 256]
	LIF	↑	Leukemia inhibitory factor (LIF) is an IL-6 class cytokine involved in controlling stem cell pluripotency, differentiation, bone metabolism, and inflammation. LIF signaling activates the JAK/STAT, MAPK, and PI3K pathways. This pleiotropic cytokine elicits a varied response in different cell types	[257]
	POSTN	↑	Periostin (POSTN) is an extracellular matrix protein that acts as a structural molecule of the bone matrix and a signaling molecule that stimulates osteoblasts through integrin receptors and the Wnt/β-cat pathway. POSTN is secreted in muscles during regeneration and differentiation	[258]
	FGF21	↑	Fibroblast growth factor 21 (FGF21) is a hormone produced by several tissues that controls various metabolic pathways. Muscle-derived FGF21 acts as a stress-induced myokine, found to promote muscle atrophy and bone loss	[259]
Muscle—microvasculature	NOS, NO	↓	Nitric oxide synthases (NOSs) catalyze the production of nitric oxide (NO) from L-arginine that controls, among others, vasodilation, and angiogenesis. NO also activates guanylyl cyclases (GC), which synthesize the second messenger cyclic guanosine monophosphate (cGMP), and act on its downstream targets, such as cGMP-activated protein kinase (PKG) or cyclic nucleotide-activated ion channels	[260]
	VEGF	↓	Vascular endothelial growth factors (VEGFs) are key regulators of vascular development and of blood vessel function. Binding of VEGF to the VEGF receptor initiates the downstream signaling cascade and ultimately results in cell proliferation, migration, and the three-dimensional arrangement to form a vascular tube	[261]
	miR-206	↑	Micro-RNA miR-206 is expressed specifically in skeletal muscles. miR-206 impedes cell proliferation and promotes SC and myoblast differentiation via posttranscriptional regulation of gene expression, boosting muscle regeneration and growth	[262]
	HIF-1α	↓	HIF-1α is a regulatory subunit of hypoxia-inducible factor-1 (HIF-1), an oxygen-dependent transcriptional activator. Target genes of HIF-1 are related to angiogenesis, cell proliferation and survival	[263]
Muscle—neuron	MuSK	↓	Muscle-specific kinase (MuSK) is a transmembrane tyrosine kinase that forms a multiprotein complex localized in the postsynaptic sarcolemma. In response to neural agrin signaling, autophosphorylation of MuSK drives intracellular signaling cascades to coordinate the local synthesis and assembly of synaptic proteins. It results in the reorganization of the cytoskeleton and the recruitment of AChR-binding scaffolding proteins to aggregate AChRs	[160]
	AChR	Defects in clustering	Acetylcholine receptors (AChRs) are ligand-gated ion channels that open upon acetylcholine binding and induce postsynaptic depolarization. AChR clustering is necessary for the proper functioning of the neuromuscular junction	[264]
	Utrophin	↑	Utrophin is a dystrophin homolog. Similar to dystrophin, utrophin presents mechanical functions and forms a signaling hub as a scaffold for various proteins. The upregulation of utrophin gene (*UTRN*) is one of the potential strategies to treat DMD	[265]
Muscle satellite cells	MARK2	↓	Microtubule affinity regulating kinase 2 (MARK2) is a serine/threonine-protein kinase that is an important regulator of cell polarity. MARK2 modulates microtubule network via phosphorylation and inactivation of microtubule-associating proteins	[46, 266]
	PARD3	Loss of polarization	Partitioning defective protein 3 (PARD3) is a part of Par complex built of atypical Protein Kinase C (aPKC)/Bazooka (Baz, PARD3)/Par-6. The Par complex determines cell polarity and asymmetric cell division. Opposite localization of Par complex and MARK2 defines the apicobasal axis	[46, 266]
	Autophagy pathways	↓	The autophagy pathway is a conserved cellular process of degradation of intracellular components that include soluble or aggregated proteins, organelles, macromolecular complexes, and foreign bodies. The formation of an autophagosome that ultimately fuses with a lysosome is driven by the cooperation of multiple factors	[267]
	Notch	↑	Notch signaling is a conserved pathway of cell–cell communication. The Notch receptor is localized on the signal-receiving cell, while ligands are located on the neighboring signal-sending cell. The effect of Notch signaling depends on the cellular context and can influence differentiation, proliferation and apoptotic cell fates	[268, 269]
	p38γ/Carm1	Mislocalization	Mitogen-activated protein kinase (MAPK) p38γ regulates SC fate through phosphorylation of Carm1, which further controls epigenetic induction of *Myf5* expression during asymmetric SC division	[9]
	FGF2	↑	Fibroblast growth factor 2 (FGF2) is one of the FGFs that regulate SC function via activation of ERK MAPK, p38 MAPKs, PI3 kinase, PLCg and STATs. SCs express FGR receptors to detect FGF2 produced by myofibers, fibroblasts and satellite cells	[270]

TLRs are expressed on the surface of immune cells and cells unrelated to the immune system, including myofibers. Activation of TLR signaling initiates cascades of molecular events that trigger the synthesis and secretion of cytokines and other proinflammatory factors necessary for both innate and adaptive immune responses [75, 76]. Cytokines are small proteins (~ 5–20 kDa) released by a broad range of cells. Their cell-surface receptors are located on various types of cells, and following binding, cytokines activate a cascade of intracellular signaling, resulting in up- or downregulation of a number of genes, although the final effect is cell-type specific. Depending on their influence on the inflammatory process, they can be divided into pro- and anti-inflammatory cytokines. Specifically, families of interleukin (IL)-1, IL-17, tissue necrosis factor-α (TNF-α), and interferons belong to the first group, whereas anti-inflammatory signaling is driven by families of IL-10 and IL-12 cytokines [77] (Table 1). Interestingly, some of them, e.g., IL-6, can have a pleiotropic effect on muscle regeneration, i.e., IL-6 interaction with its soluble receptor mediates pro-inflammatory response while binding to its membrane-bound receptor triggers a cascade of regenerative or anti-inflammatory cytokine action [78, 79] (Table 1). At the time that the mechanisms of innate immunity are already active, the major histocompatibility complex (MHC) proteins present antigens and initiate an adaptive immune response involving the action of T and B cells. Chronic inflammation can adversely affect tissues and organs, and the current data indicate that continuous stimulation of the immune system sustained by the ongoing degeneration and regeneration of myofibers adversely affects the condition of DMD patients.

**Fig. 3:**
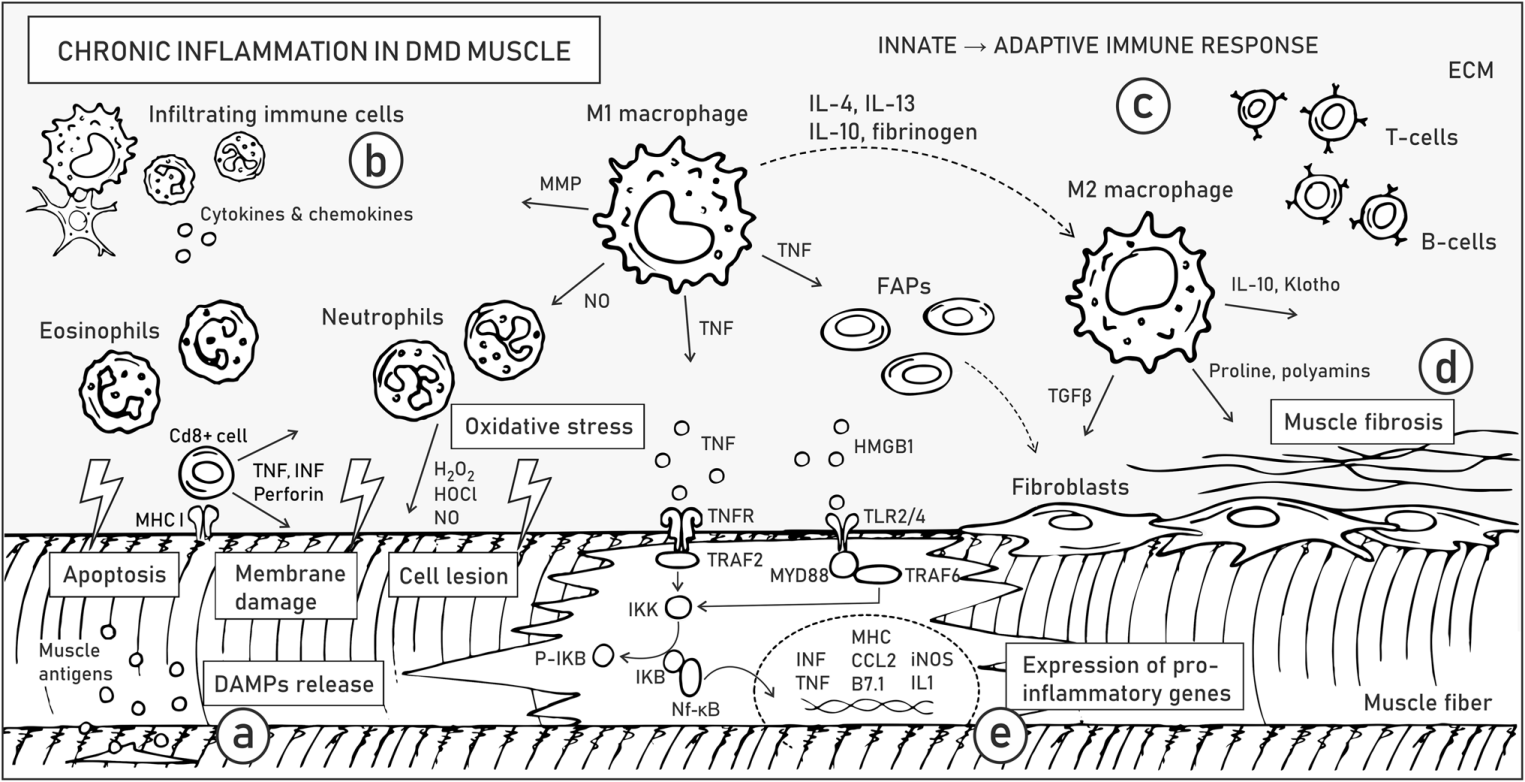
Chronic inflammation in DMD skeletal muscles. Pathological alterations related to chronic inflammation are listed in boxes. Arrows illustrate the release of various factors, while dashed line arrows depict the transformation of one type of cell to another. Molecular mechanisms underlying ongoing processes of sarcolemma instability and DAMP release **a**, activation of the innate immune response **b**, development of adaptive immune response **c**, muscle fibrosis **d**, and inter- and intracellular signaling driving inflammatory response **e** are described in the text. Abbreviations: extracellular matrix (ECM), damage-associated molecular patterns (DAMPs), major histocompatibility complex I (MHCI), matrix metalloproteases (MMP), fibroadipogenic progenitors (FAPs), interferon γ (INFγ), tissue necrosis factor (TNF), receptor of TNF (TNFR), TNFR Associated Factor 2 (TRAF2), IκB kinase (IKK), IKB, phosphorylated IKB (P-IKB), nuclear factor-κB (NF-κB), high-mobility group box 1 (HMGB1), Toll-like receptors (TLR2/4), TNFR associated factor 6 (TRAF6), myeloid differentiation primary response 88 (MYD88), interleukin (IL), transforming growth factor-β (TGFβ)

Membrane instability and leakage of cytoplasmic content into the extracellular matrix initiate chronic activation of the innate immune system in DMD muscles (Fig. 3a). DAMPs, including nucleic acids, heat shock proteins (HSPs), high-mobility group box 1 (HMGB1) proteins, and ROS, are released by destroyed fibers and activate TLRs, e.g., TLR2/4 in myofibers (Fig. 3a). Signaling from TLRs and the IL-1 receptor activates a downstream adaptor protein, myeloid differentiation primary response 88 (MyD88) (Table 1). MyD88 activates IL-1R-associated kinase (IRAK) family kinases, which in turn trigger mitogen-activated protein kinases (MAPKs) and proinflammatory transcription factors, such as nuclear factor-κB (NF-κB; Fig. 3e) and activator protein 1 (AP-1). Importantly, Gallot et al. (2018) showed that inhibition of TLRs or MyD88 in dystrophic mice alleviates the disease symptoms and reduces inflammation [80] (see also “Disrupted signaling in satellite cells”).

After muscle injury, the tissue is infiltrated by innate immune system cells. Among others, (1) CD8 + cytotoxic T lymphocytes induce apoptosis of myofibers through a perforin-mediated pathway, (2) eosinophils release lytic substances and (3) inflammatory M1 macrophages (activated by proinflammatory cytokines, such as IFN-γ) produce nitric oxide (NO) and inflammatory cytokines and participate in direct phagocytosis (Fig. 3b). Macrophage-derived NO further induces muscle fiber lysis and tissue damage, which stimulates neutrophils to release free radicals [81]. Local elevations in oxidative stress aggravate the pathology of DMD muscles, partially through changes in signal transmission [82]. Proinflammatory cytokines trigger constitutive expression of MHC class I and II on muscle cells, which attract T and B cells, consequently inducing an adaptive immune response [83] (Fig. 3c). Meanwhile, promoted by IL-10, IL-4 and IL-13, modulatory M2 macrophages gradually replace M1 macrophages. M2 macrophages release anti-inflammatory cytokines, such as IL-10, IL-4 and transforming growth factor-β (TGF-β) [81] that alleviate muscle injury (Fig. 3b) (Table 1).

The various inflammatory molecules produced by macrophages include matrix metalloproteases (MMPs), which are known to promote inflammation and interstitial fibrosis (Fig. 3d) (Table 1). In particular, high levels of MMP-9 were detected in numerous muscle conditions, including the skeletal muscles of DMD mouse models and patients [84]. Moreover, the level of MMP-9 correlated with disease severity [85]. Hindi et al. showed that inhibition of MMP-9 boosts the promyogenic M2 phenotype while diminishing the percentage of M1 macrophages in *mdx* mice [84]. Accordingly, MMP-9 downregulation reduced the levels of the inflammatory cytokines IFN-γ and IL-6 and increased the activity of IL-4 (involved in adaptive immunity). The reduced inflammation was also confirmed by the reduced levels of the proinflammatory transcription factors NF-κB and AP-1 (Table 1). Furthermore, enhanced proliferation of SCs and improved regeneration were observed [84].

During DMD muscle degeneration, M1 macrophages and myogenic cells are among the main sources of the proinflammatory factor TNF-α (Fig. 3b) (Table 1), which is suggested to be the major death ligand driving necrosis and programmed necrosis (necroptosis) and is linked to the activation of receptor-interacting protein kinase-1 (RIPK3) signaling [86]. Elevated TNF-α levels also contribute to the aggravation of inflammation via the induction of NF-κB signaling, which triggers the expression of proinflammatory genes and the synthesis of cytokines and chemokines. The Acharyya group demonstrated upregulation of IκB kinase/NF-κB (IKK/NF-κB) signaling in DMD and revealed that reduction of NF-κB or its upstream activator IKK improves the pathology and muscle function in *mdx* mice [87]. In the same animal model, Yang et al. showed that AAV-mediated shRNA knockdown of the p65 subunit of NF-κB has an anti-inflammatory effect [88]. Genetic reduction of p65 levels also diminished chronic inflammation and improved DMD muscle physiology [89], and by targeting RANKL (the receptor activator of nuclear factor NF-κB ligand), it alleviated the pathology by increasing the proportion of M2 macrophages and reducing muscle edema and fibrosis [90]. Furthermore, anti-RANKL treatment also increased the mechanical properties of bone in dystrophic mice [90]. Overall, inhibition of NF-κB may be a promising therapy for DMD, especially when combined with gene therapy designed to restore dystrophin expression.

The alleviating role of M2 macrophages is also linked to the increased synthesis and secretion of Klotho, a transmembrane protein whose extracellular domain can be cleaved and released (Fig. 3b) (Table 1). Klotho functions as a promyogenic circulating hormone [91] that activates the proliferation and growth of muscle cells. Accordingly, Klotho expressed by leukocytes positively influences the number of SCs in dystrophic muscle [91]. The expression of Klotho is significantly decreased in DMD tissues; the diminished activity of the Klotho pathway in muscle cells is attributed to epigenetic changes associated with oxidative stress in dystrophic muscle; and overexpression of *Klotho* in macrophages counteracts the expression of profibrotic genes and reduce the pathology of DMD [92]. Under physiological conditions, Klotho diminishes TGF-β expression and thus prevents muscle fibrosis, whereas in DMD patients and *mdx* mice, TGF-β remains activated and consequently promotes muscle fibrosis.

### Muscle–bone relation

**Fig. 4:**
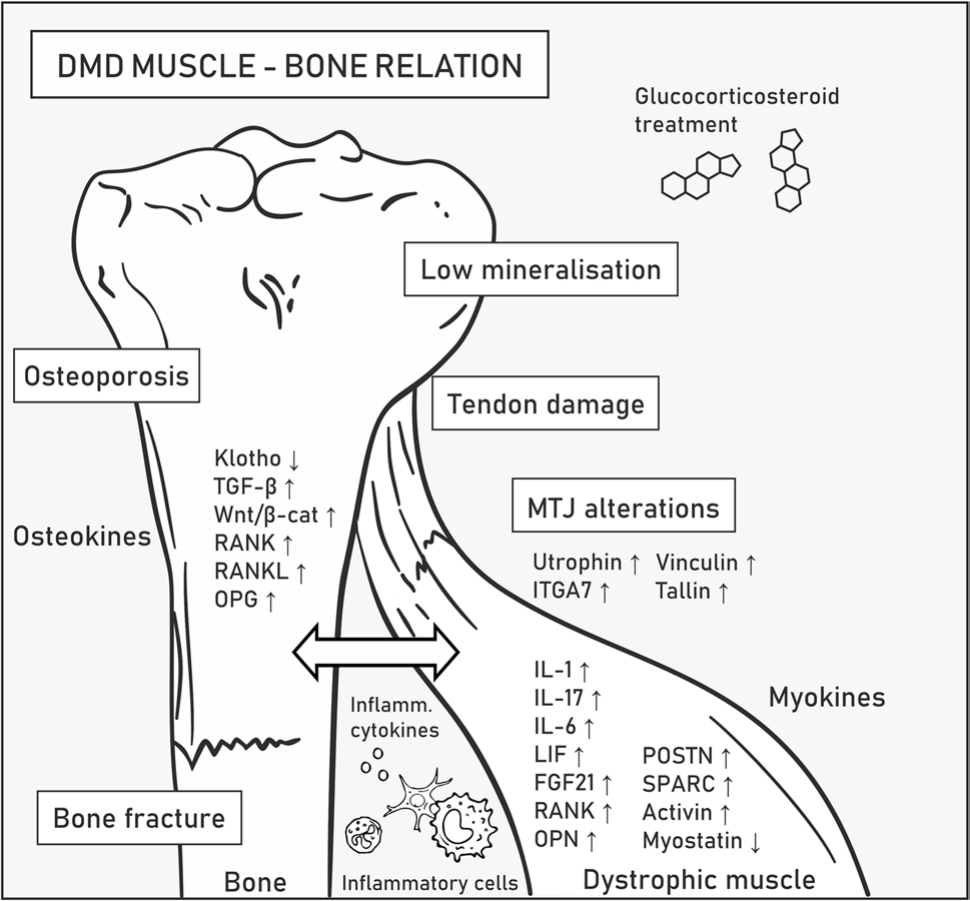
Muscle–bone relation in Duchenne muscular dystrophy. DMD symptoms related to bones and tendons associated with skeletal muscles are listed in boxes. Molecular mechanisms underlying pathogenesis in the muscle–bone relation are described in the text. Abbreviations: transforming growth factor-β (TGF-β), Wnt/β-catenin (Wnt/β-cat), receptor activator of NF-κB (RANK), RANK ligand (RANKL), osteoprotegerin (OPG), interleukin (IL), leukemia inhibitory factor (LIF), fibroblast growth factor 21 (FGF21), periostin (POSTN), osteonectin (SPARC), integrin α7 (ITGA7)

Low bone mineral density and fragility in DMD are symptoms that accompany muscle degeneration caused by inflammation and alterations in signaling and cell-to-cell communication [93]. Pathological fractures of long bones and vertebrae significantly impact mobility and decrease the patients’ quality of life [94]; moreover, the standard long-term therapeutics for DMD, i.e., glucocorticosteroids, increase the risk of osteoporosis [95]. DMD mouse models also display a decline in bone biomechanical properties and healing capacity, spinal deformity, and spontaneous ossification in muscles [96–98]. Muscle-derived myokines, osteokines released by the bone, and inflammatory cytokines trigger common signaling pathways, thus providing a functional connection between the cells organized into the muscle–bone unit [71, 93] (Fig. 4).

IL-6 is a pro-osteoclastogenic and pro-myogenic cytokine released by tissues with essential functions in bone homeostasis [93] and myogenesis [99]. IL-6 is upregulated in DMD patients and *mdx* mice compared with healthy controls, and it can mediate bone degradation and elevate the level of IL-10 that switches pro-inflammatory M1 macrophages to anti-inflammatory M2 macrophages, which drives an anti-inflammatory response. The levels of IL-10 and its receptor are also higher in DMD muscles [71]. However, as previously mentioned, IL-6 shows a pleiotropic phenotype during muscle repair, and despite its anti-inflammatory and regenerative effects, it can also aggravate inflammation [100]. Specifically, Pelosi et al*.* showed that forced expression of IL-6 exacerbates muscle pathology while its receptor blockade has the opposite outcome in *mdx* mice [79, 100].

LIF is an example of an IL-6 class cytokine involved in bone remodeling and shows higher expression in dystrophic muscles (Table 1). In particular, the study by Matsushita et al. showed that LIF inhibits osteogenesis via the STAT3/SOCS3 signaling pathway [101]. Bone weakness in DMD patients is also driven by osteopontin (OPN), a well-known inhibitor of bone mineralization and a factor that promotes fibrosis in dystrophic muscles [102] (Table 1). This osteokine, among others, is produced by inflammatory cells, e.g., macrophages; therefore, its expression level greatly increases during inflammation. Proinflammatory cytokines engaged in bone homeostasis, such as IL-1 and IL-17, are also elevated in DMD [93, 103, 104]. Thus, potential therapeutic treatments based on reducing the level of various molecules driving inflammation to alleviate inflammation in diseased muscles can be envisioned. Such approaches, however, require in-depth knowledge of the remote effects of these compounds in various organs. For example, IL-15 treatment is potent enough to improve the pathophysiology of dystrophic muscle in *mdx* mice [105] but was also shown to stimulate osteoclast differentiation, reduce the number of both osteoclasts and osteoblasts in bone marrow, and increase the bone mineral content [71].

Under pathological conditions of dystrophin loss-mediated muscle fiber degeneration and decreased Klotho levels, TGF-β is released in high amounts from the bone matrix, which contributes to muscle weakness. TGF-β promotes myofibroblast differentiation and increases tissue fibrosis by stimulating the canonical Wnt/β-catenin pathway that regulates bone homeostasis and myogenesis (Table 1). In particular, Wnt/β-catenin decreases osteoclast differentiation by promoting the synthesis and secretion of osteoprotegerin (OPG), an osteoclastic inhibitor [106]. Although therapeutic overexpression of *Klotho* indeed improves the functioning of muscles [92], it was also shown to inhibit mineralization and osteogenic activity in cultured osteoblastic cells, presumably due to decreased Wnt/β-catenin and downregulation of OPG [71]. OPG is produced by osteoblasts and prevents the interaction of receptor activator of NF-κB (RANK), which is located on preosteoclastic cells, with RANKL, thus inhibiting the NF-κB pathway [90] (Table 1). RANK is also present in the sarcolemma of muscle fibers, while RANKL and OPG were found to be expressed in the myoplasm and can be secreted from myofibers, indicative of the bidirectional interaction between both tissues. Moreover, RANK/RANKL signaling is disrupted in *mdx/utrn*^+/−^, with significantly upregulated RANKL and RANK protein levels observed in *mdx/utrn*^+/−^ muscle samples [90]. Overall, the current data indicate that the RANK/RANKL/OPG pathway may be an important platform for muscle–bone crosstalk, which is dysfunctional in DMD patients.

In addition to RANK and RANKL, the expression of many other bone-regulating myokines is altered in DMD, and they include the extracellular matrix proteins periostin (POSTN) and osteonectin (also known as SPARC), which are upregulated in DMD muscles and play important roles in bone remodeling as well as maintaining bone mass and quality by a variety of mechanisms [104] (Table 1). Furthermore, fibroblast growth factor 21 (FGF21) was reported to be elevated in DMD muscles [104] and to negatively regulate bone homeostasis by potentiating peroxisome proliferator-activated receptor gamma (PPAR-γ) activity, resulting in adipogenesis stimulation and osteogenesis inhibition from bone marrow stem cells. Increased FGF21 levels were also found to indirectly promote osteoclastogenesis, presumably by increasing the RANKL/OPG ratio [107] (Table 1).

Activin and myostatin are growth factors that belong to the TGF-β superfamily, and they are known to have negative effects on muscle and bone mass [108, 109]; therefore, their downregulation might be considered a therapeutic approach to prevent muscle wasting and bone degeneration in DMD patients [71]. Follistatin modulates bone metabolism presumably via activin and myostatin signaling, and follistatin-based gene therapy was shown to have positive effects on muscles [110–112]. However, myostatin seems to have a positive impact on tendons. Specifically, in myostatin-deficient mice, tendons have a stiff, brittle and hypocellular phenotype. Stiffness of tendons would worsen the course of DMD and lead to higher sensitivity to contraction-induced injury [113].

Tendons allow movement by providing physical muscle interaction with the skeleton. In DMD patients, the ongoing cycles of myofiber degeneration and regeneration increase fibrosis and results in pseudohypertrophy of certain muscles, especially in the early stages of the disease [114], which results in excessive strain on tendons and in their shortening and contracture (often the case with the Achilles tendon) [115]. The elastic properties of dystrophic tendons are also compromised due to the presence of a higher number of dead cells and collagen concentration as well as the reduction in proteoglycans and infiltration of inflammatory cells [115, 116]. The architecture of myotendinous junctions (MTJs), i.e., specialized structures located on the muscle–tendon interface, is also affected in DMD. Specifically, dystrophic MTJs contain a reduced number of sarcolemmal folds [43, 117], with the compensatory upregulation of a number of proteins, including utrophin, α7 integrin, vinculin, and talin [118, 119].

### Muscle–microvasculature relation

**Fig. 5:**
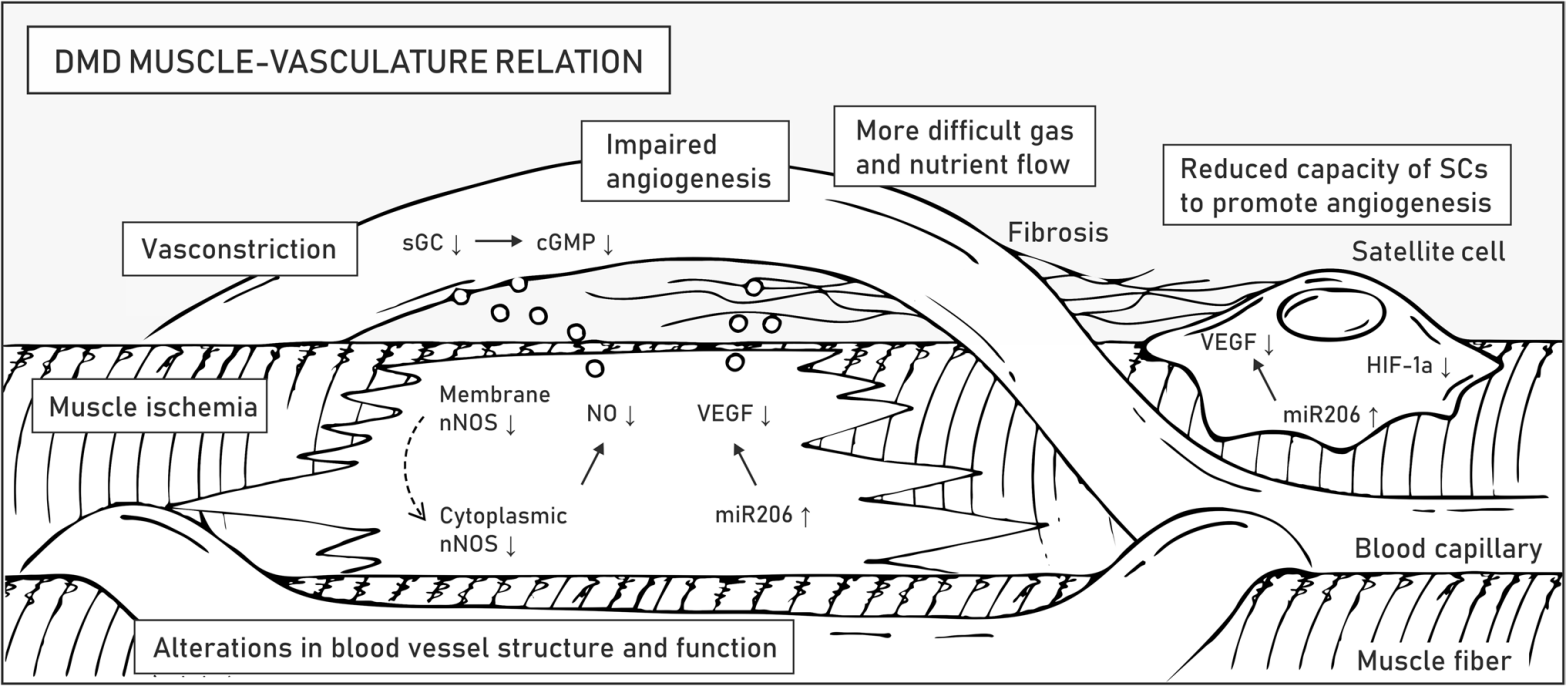
Muscle–vasculature relation in DMD. Symptoms related to the microvasculature of striated muscles are listed in boxes. Molecular mechanisms underlying pathogenesis in the muscle-vasculature relation are described in the text. Abbreviations: neuronal nitric oxide synthase (nNOS), soluble guanylyl cyclase (sGC), cyclic guanosine monophosphate (cGMP), vascular endothelial growth factor (VEGF), hypoxia-inducible factor-1α (HIF-1α)

Skeletal muscle is a highly vascularized tissue. The presence of necrotic muscle bundles due to local ischemia was one of the first described causes of muscle weakness in DMD before the discovery of the dystrophin gene [120]. Indeed, proper vascularization is obligatory for normal functioning and regeneration of muscle tissue. In addition to myofibers and satellite cells, *DMD* is expressed in vascular smooth muscle and endothelial cells, and a lack of full-length dystrophin directly affects the formation of blood vessels [47]. Impaired signaling between the muscle tissue and surrounding capillaries also aggravates the phenotype of the patients (Fig. 5).

The vascular hypothesis of DMD indicates the critical role of the neuronal nitric oxide synthase (nNOS) pathway [72, 121] (Table 1). In healthy myofibers, the muscle-specific isoform of nNOS (nNOSμ) is anchored to the sarcolemma by binding with dystrophin spectrin-like repeat 17 through α-syntrophin [122] (Fig. 1). nNOSμ-derived nitric oxide (NO) diffuses to neighboring capillaries, where it increases the concentration of cyclic guanosine monophosphate (cGMP) and counteracts vasoconstriction caused by norepinephrine released from sympathetic nerves. Dystrophin deficiency causes displacement of nNOSμ from the sarcolemma to the cytoplasm, where its amount is also greatly reduced [123, 124]. Consequently, DMD myofibers are more susceptible to functional muscle ischemia during exercise and injury due to a reduction in paracrine signaling [123, 124]. The ischemic phenotype inspired multiple studies aimed at targeting the nNOS-NO pathway in DMD patients based either on the expression of dystrophin minigenes to provide the nNOSμ binding site, direct overexpression of the nNOS gene, supplementation with L-arginine (nNOS substrate), delivery of NO-donating drugs, or inhibition of phosphodiesterase 5A (PDE5A) to prolong the half-life of cGMP [72].

Therapeutic strategies grounded on modulation of the nNOS pathway are oriented on increasing vasorelaxation of the vasculature to enable perfusion of diseased muscles. A different approach to obtaining the same effect is to enhance the density of the microvasculature network by boosting angiogenesis [47, 124]. Vascular endothelial growth factor (VEGF) is a glycoprotein synthetized and secreted by myofibers and SCs in muscle tissue, and it plays a crucial role in this process, mainly via the VEGFR-2 pathway [125] (Table 1). VEGF stimulates the proliferation, migration, and survival of endothelial cells upon binding to vascular endothelial growth factor receptors (VEGFRs) [125] and has antiapoptotic properties toward myogenic cells [126]. Importantly, VEGF levels increase during exercise in healthy muscles [47], a process that is abrogated in DMD patients because their physical activity is reduced and associated with tissue damage.

As *mdx* muscles undergo chronic degeneration–regeneration routes, a strong upregulation of microRNA-206 (miR-206), one of the key regulators of myogenesis synthesized in both muscles and SCs, is observed (Table 1). Apart from its role in muscle repair [127], miR-206 also represses the expression of the VEGF gene in myofibers and therefore negatively impacts angiogenesis [47]. Treatment strategies targeting VEGF signaling include inhibition of VEGFR-1 (a negative regulator of angiogenesis, competing for VEGF with VEGFR-2), direct delivery of VEGF, administration of rAAV vectors carrying the coding sequence of VEGF, or in vivo transplantation of muscle-derived stem cells (MDSCs) with upregulated VEGF. Nevertheless, it is important to note that a high dosage of VEGF can contribute to a profibrotic response and lead to serious adverse effects, e.g., endothelial cell-derived vascular tumors [47]. As an alternative approach, heme oxygenase 1 (HO-1) was found to regulate blood vessel formation and angiogenesis induced by VEGF and stromal cell-derived factor 1 (SDF-1) [128, 129], and such effects in addition to other aspects of HO-1 signaling (see “Disrupted signaling in satellite cells”) underlie the rationale for the therapeutic modulation of HO-1 levels to ameliorate DMD pathology. A treatment based on a different growth factor, angiopoietin 1 (ANG1), combined with VEGF or alone, was also proposed. Notably, administration of ANG1 was shown to enhance muscle perfusion and slow the progression of fibrosis [130].

Signaling crosstalk between capillaries and SCs that are localized in their close proximity may also influence angiogenesis and muscle regeneration. In particular, SCs derived from 12-month-old *mdx* mice showed a reduced ability to promote angiogenesis in vitro [131], presumably due to the age-related lower levels of proangiogenic VEGF and hypoxia-inducible factor-1α (HIF-1α) [131] (Table 1). Other reports indicate that in young dystrophic animals, showing intensive degeneration and regeneration cycles, angiogenesis is not altered or even enhanced [132, 133]. Interestingly, SCs also seem to be influenced by the muscle vasculature, e.g., Verma et al. showed that their direct interaction may influence the self-renewal and quiescence of SCs via Notch signaling [134] (Table 1). Increased distance between blood vessels and myofibers could also slow gas exchange and impede reciprocal signaling as a result of fibrosis-associated changes in ECM [135]. Latroche et al. compared the microvasculature of 3- and 12-month-old *mdx* mice that differed in the content of the fibrotic tissue [136], and they identified very severe alterations of the microvascular network structure with reduced perfusion only in older mice. Overall, the current research data clearly indicate that hindered muscle vascularization contributes to the pathophysiology of DMD.

An additional issue associated with muscle-microvasculature interactions is the therapeutic potential of pericytes that localize underneath the basal lamina of small vessels [137]. Pericytes differentiate with high efficiency into skeletal muscle cells in vivo and those isolated from one biopsy can be expanded in vitro to amounts sufficient to treat a pediatric patient [138]. Pericytes are also suitable for systemic delivery because they can cross the vessel wall and are easily transducible with viral vectors [137, 138]. In contrast, SC-derived cells do not efficiently cross the endothelial layer [139, 140] and are challenging to transduce with viral vectors [141, 142].

### Muscle–neuron relation

Neuromuscular junctions (NMJs) are specialized regions where terminal buttons of motor neurons contact muscle fibers to form chemical synapses. The signal is transmitted by a small molecule neurotransmitter, acetylcholine (ACh), which, following release from motoneurons, binds ACh receptors (AChRs) on the postsynaptic sarcolemma (called motor end-plate) (Table 1). This interaction enables ion flow across the sarcolemma that leads to local depolarization and induction of an action potential that travels across the myofiber membrane, eventually leading to muscle contraction. The structural organization of the sarcolemma and clustering of AChRs are important factors in efficient signal transmission, signified by the fact that some conditions, such as exercise [143, 144], aging [145], muscle or nerve injury, and lack of associated proteins [146], result in structural and functional alterations.

**Fig. 6:**
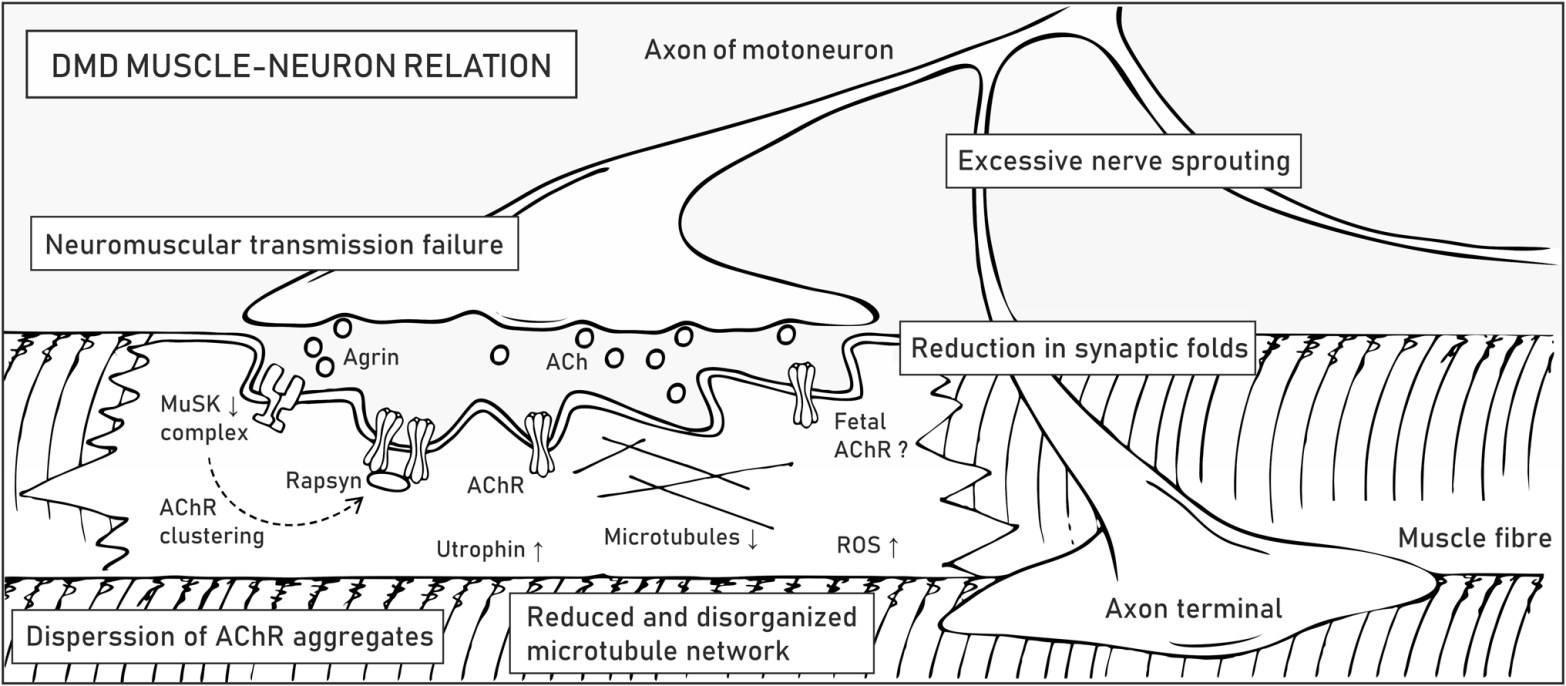
Pathological alterations in dystrophic NMJs. DMD symptoms at NMJs are listed in boxes. The molecular mechanisms underlying the pathogenesis of NMJs are described in the text. Abbreviations: muscle-specific kinase (MuSK), acetylcholine (ACh), acetylcholine receptor (AChR), reactive oxygen species (ROS)

DAPC accumulates at the postsynaptic membrane of NMJs, where it is required for synaptic homeostasis [29]. *Mdx* mice show structural abnormalities that include loss in the number and depth of synaptic folds in the motor end-plate, NMJ fragmentation (more scattered AChR clusters), and excessive nerve sprouting [146, 147] (Fig. 6). Such morphological alterations entail profound functional consequences, as revealed by reduced end-plate potential (EPP) and depressed safety factor (the amplitude by which the EPP exceeds depolarization threshold) [148]. Utrophin is also present at the NMJs of healthy individuals and in higher amounts in DMD muscles [149], where it presumably compensates for the absence of dystrophin [64]. *Utrn*^−/−^ mice have a lower number of AChRs and reduced folding of the postsynaptic membrane; nonetheless, they show no signs of weakness, which suggests that utrophin is not essential but undoubtedly contributes to the organization and maintenance of NMJs [150]. This conclusion is further corroborated by qualitative assessment of NMJs in various DMD mouse models, e.g., *mdx*, *mdx/utrn*^+/−^, and *mdx/utrn*^*−/−*^, which revealed the most severe damage in mice devoid of both dystrophin and utrophin. However, AChR area fragmentation at NMJs was observed for all three DMD mouse models, with no significant difference among them [151].

Because disruption of NMJs is one of the main symptoms of DMD, therapeutic strategies are assessed to restore proper communication between nerves and muscles. Although estimates of how much dystrophin is required for the therapeutic effect depend on various factors, e.g., quantification method or reference samples [152, 153], the current results indicate that even a fraction of the normal dystrophin levels could be beneficial in dystrophic muscles. The proper formation and functioning of NMJs might require higher dystrophin amounts, estimated to range between 19 and 50% of the normal content [154]. On the other hand, utrophin upregulation ameliorates muscle pathology in *mdx* mice and leads to increased AChR clustering and improved morphology of NMJs [155], which shows that either utrophin- or dystrophin-based therapies could be beneficial for the treatment of DMD [64]. In their review, Ng and Ljubicic [148] concisely reported observations regarding the morphology and electrophysiology of NMJs obtained since the 1970s and concisely presented potential therapeutic strategies targeting neuromuscular transmission in dystrophic muscles.

Sequence and structural similarity of dystrophin and utrophin enable the formation of protein complexes characterized by partial functional interchangeability [40]. Both DAPC and utrophin-associated protein complex (UAPC) contain two subunits of dystroglycan that are of special importance at NMJs [156]. α-Dystroglycan is present at the cell periphery on the outer surface of the sarcolemma, where it interacts with a number of proteins, including agrin, a signaling molecule released by the nerve terminal that initiates clustering of AChRs on the postsynaptic membrane, as well as laminin, collagen, entactin, and perlecan. Through the interaction with the latter, α-dystroglycan associates with acetylcholinesterase, which is important in acetylcholine breakdown [157]. Moreover, the transmembrane β subunit of dystroglycan stabilizes the structure of NMJs, thereby facilitating the formation of the cytoskeletal network needed for clustering and stabilization of AChRs at the sarcolemma [156].

α- and β-Dystroglycan are also necessary for the proper organization of other membrane protein assemblies, including the multiprotein complex associated with muscle-specific kinase (MuSK) (Table 1). MuSK is distributed in the postsynaptic sarcolemma, where it plays a pivotal role in AChR clustering and end-plate maintenance [158]. In DMD, loss of dystroglycans leads to reduced levels of MuSK, which presumably directly impacts NMJ morphology and function. Consistent with this finding, *Musk*^*−/−*^ mice cannot move and breathe and die at birth due to neuromuscular transmission loss [158]. Additionally, conditional inactivation of MuSK in adult mice resulted in the absence of AChRs, disassembly of the postsynaptic organization, severe muscle weakness and premature death [159]. Other studies have shown that the MuSK complex drives the aggregation of AChRs in response to agrin binding, which initiates the autophosphorylation of MuSK and the formation of a stable and active MuSK complex. This process in turn initiates a downstream signaling cascade that results in recruitment of rapsyn to AChRs and stabilization of the AChR clusters through the DAPC/UAPC linkage [160, 161] (Fig. 6). Rapsyn is also involved in recycling and lifetime regulation of AChRs, a process that is under control of cAMP/PKA signaling, which is shown to be disturbed at dystrophic NMJs [162].

Loss of dystrophin and agrin signaling at the NMJ alters the organization of the microtubule network [66, 163]. Importantly, despite being upregulated at NMJs, utrophin cannot restore the microtubule lattice in dystrophic muscles, as it does not contain the microtubule-binding domain observed for dystrophin (Fig. 1) [66]. Microtubules maintain the right shape of the cell and allow for positioning of organelles and alterations in their network could affect mitochondrial density and function, thereby leading to excessive production of ROS [147, 164]. Another important aspect linked to DMD pathology is the presence of embryonic-type AChRs, as revealed in regenerating muscle fibers of *mdx* mice [165]. Specifically, Pijl et al. speculated that longer open embryonic-type AChRs in constantly regenerating dystrophic muscles may induce Ca^2+^ overload and contribute to myofiber focal necrosis by activating Ca^2+^-dependent proteases [151].

### Disrupted signaling in satellite cells

SCs are muscle stem cells that are localized along the myofiber between the sarcolemma and the surrounding ECM (also termed basal lamina) and responsible for postnatal tissue growth and regeneration [166]. In the process of muscle repair, SCs are supported by other cell types that compose the regenerative milieu, including infiltrating immune cells (macrophages, eosinophils, regulatory T-cells, neutrophils), vascular endothelial cells, pericytes, fibroblasts, and FAPs, also called mesenchymal stromal cells [167]. In particular, the latter was recently reported to play crucial roles in muscle repair and maintenance. Experiments in mice revealed that paracrine signaling between SCs and FAPs supports myogenesis by influencing the proliferation and differentiation of SCs [167] while depletion of FAPs results in lessening of the SC pool, regenerative deficit, and muscle atrophy [73]. Some interstitial cells may also have myogenic potential, including myoendothelial [168], CD133 + [169, 170], PW1 interstitial [171], muscle-derived stem cells (MDSCs) [172, 173], Twist2 + progenitor [174] and muscle side population cells [167, 175]. Nonetheless, it is important to note that they are unable to regenerate muscle in the absence of SCs [176].

Quiescence of SCs in resting muscle is maintained by expression of the paired box 7 (*Pax7*) gene controlled by the Notch pathway, which is activated by binding of Notch ligands distributed at the sarcolemma to Notch receptors located on the SC membrane [177] as well as various epigenetic mechanisms [11]. Specifically, nonmethylated Pax7 protein limits the expression of myogenic factor 5 (*Myf5*) while its methylation enables recruitment of epigenetic machinery to the *Myf5* promoter and in turn SC activation, division and differentiation into myotubes [178]. Importantly, SCs can divide in two ways: symmetrically and asymmetrically [179]. While symmetric divisions (occurring parallel to the contiguous myofiber in a planar orientation) preserve the stem cell pool by generating two identical daughter cells, asymmetric divisions (occurring in apicobasal orientation, perpendicular to myofiber) result in two different daughter cells, with one of them returning to the state of quiescence and the other entering the myogenic pathway. The expression of *Myf5* is characteristic of myoblasts, while quiescent cells remain Myf5-negative and a direct contact with the basal lamina on one side and the myofiber sarcolemma on the other is a suspected factor determining the polarity of dividing SCs [180]. Notably, during muscle regeneration, SCs can also migrate to interstitial areas of the muscle, where they can proliferate and differentiate into new fibers [181, 182]. A detailed description of the intrinsic and extrinsic mechanisms regulating SC activity can be found elsewhere [178, 179, 183].

**Fig. 7:**
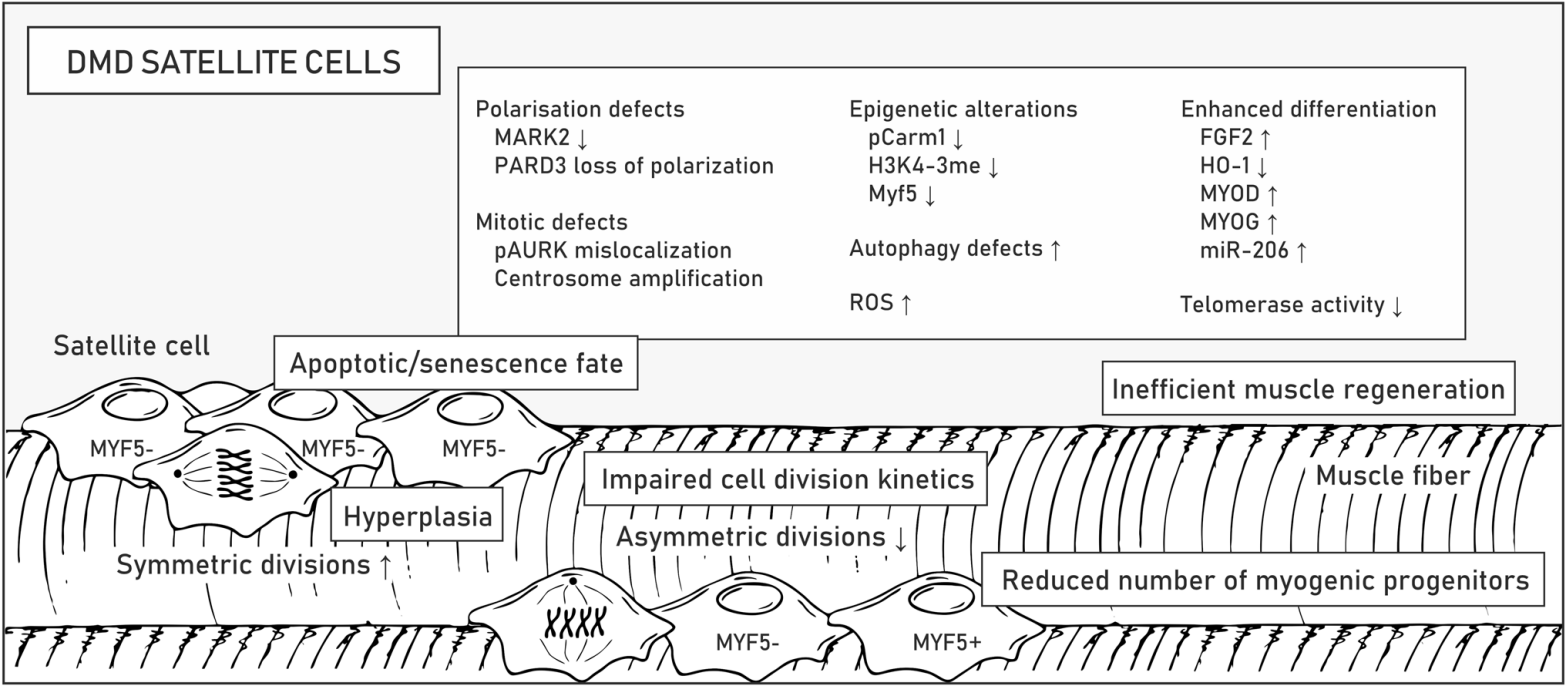
Altered functioning of DMD satellite cells. Pathological alterations related to satellite cell function are listed in boxes. Examples of disturbed signaling pathways, altered gene expression and epigenetic changes are shown in the boxes. Molecular mechanisms underlying pathologies in DMD satellite cells are described in the text. Abbreviations: myogenic factor 5 (Myf5), microtubule affinity regulating kinase 2 (MARK2), partitioning defective protein 3 (PARD3), phosphorylated Aurora kinase (pAURK), reactive oxygen species (ROS), fibroblast growth factor 2 (FGF2), heme oxygenase 1 (HO-1), myoblast determination protein (MYOD), myogenin (MYOG)

In healthy muscles, dystrophin distributes unevenly in activated SCs, thus providing the background for their asymmetric division and myogenic differentiation by anchoring to the plasmalemma microtubule affinity regulating kinase 2 (MARK2) and β1-syntrophin (Fig. 1), with its associated mitogen-activated protein kinase (MAPK) p38γ (also known as MAPK12) [184]. In dystrophic SCs, the greatly reduced content of MARK2 as well as affected polarization of partitioning defective protein 3 (PARD3) and localization of phosphorylated Aurora kinase A (Aurka), impair the apicobasal mitotic spindle orientation, centrosome amplification and division kinetics [46, 184] (Fig. 7; Table 1). Loss of β1-syntrophin and dysregulated p38γ signaling, on the other hand, disable transportation of Carm1 to the nucleus, without which Pax7 cannot be efficiently methylated to trigger *Myf5* transcription [185]. Carm1 not only contributes to disturbances in epigenetic control of myogenic gene expression but also induces autophagy-related and lysosomal genes as a coactivator of transcription factor EB (TFEB) via increasing dimethylation of histone H3 Arg17 [186]. Dystrophin loss-mediated Carm1 absence in the nucleus could thus explain the autophagy defects in DMD muscles in addition to persistent activation of Akt and mTOR triggering autophagy-inhibiting pathways [187, 188] (Table 1). Importantly, the failed autophagy pathway may result in the inability to clear damaged mitochondria that could drive the accumulation of ROS and SC senescence [189].

Although asymmetric division is disrupted, stem cell hyperplasia is observed in muscles of DMD patients and *mdx* mice, as demonstrated by the elevated numbers of Pax7-positive cells, presumably as a consequence of preferable symmetric SC division [190–192] (Fig. 7). This surplus of SCs, however, does not seem to contribute to muscle repair, and over time, the Pax7 + /Myf5 + muscle progenitor pool declines [46]. Apart from the proliferation deficits, factors that contribute to this reduction include susceptibility to oxidative stress, progressive telomere shortening due to insufficient telomerase activity and high muscular fibroblast growth factor 2 (FGF2) content, which leads to loss of stem cell quiescence [193] (Table 1).

The simultaneous decrease in the level of HO-1 enzyme with the upregulation of myoblast determination protein (MyoD), myogenin (Myog) and miR-206 also implies enhanced differentiation of dystrophic cells that do enter the myogenic differentiation program [194]. Repression of the HO-1-encoding *Hmox1* gene in DMD SCs is linked to downregulation of Atf3, MafK, Foxo1 and Klf2 transcription factors as well as attenuation of NO-mediated cGMP-dependent signaling [194]. More intensive differentiation of HO-1-deficient SCs and myoblasts was associated with changes in the activity of miRNA processing enzymes and, consequently, alterations in the miRNA transcriptome [194, 195]. Moreover, diminished HO-1 levels in *mdx* SCs were linked to increased content of Casp3 [194], which is known to target Pax7 and activate SC differentiation [196]. Interestingly, in myofibers and inflammatory leukocytes, the level of HO-1 is elevated in *mdx* mice compared to control mice, which presumably has an anti-inflammatory and alleviating effect. Pietraszek-Gremplewicz et al*.* showed that supplementation of differentiating dystrophic SCs with CO (one of the products of HO-1 enzyme) and NO (induces *Hmox1* expression) normalizes the differentiation of *mdx* SCs [194]. Targeting HO-1 is thus a potentially attractive therapeutic target.

Notch signaling determines many stages of muscle regeneration, including the fate of SCs, proliferation of myoblasts, and the transient inhibition of terminal differentiation of myoblasts into mature myofibers [197]. Moreover, a wide variety of Notch receptors and ligands have to be precisely regulated in a time- and space-restricted manner [197]. Both the quiescent and active states of SCs depend on Notch signaling, and the oscillation of Hes (Notch effector) was found to determine the level of MyoD [198]. In DMD muscles, Notch signaling is dysregulated. Specifically, Mu et al. showed that inhibition of Notch signaling, which is overactivated in DMD muscles and SCs, delays the depletion and senescence of muscle progenitor cells and reduces inflammation and fibrosis in *mdx/utrn*^*−/−*^ mice [199]. In contrast, the results from experiments on two mildly affected golden retriever muscular dystrophy (GRMD) dogs [200], which showed a higher content of Jagged 1 (a Notch ligand) due to increased expression of *Jag1*, indicated the beneficial effects of Notch activation [201]. Compared with the severely affected GRMD dogs, these “escapers” dogs could run, jump and more easily stand on their hindlegs and had a normal lifespan despite the absence of muscle dystrophin [200, 201]. A transcription factor binding site analysis revealed that mildly affected dogs contain a novel myogenin binding site in the *Jag1* promoter, which resulted in a myogenin-associated increase during muscle regeneration [201]. Experiments based on the *sapje* zebrafish, another severe DMD animal model, and an in vitro analysis of GRMD muscle cells from biopsies confirmed the beneficial effects of *Jag1* overexpression. Other studies indicate that in addition to the muscle growth and expansion of the satellite cell pool, Jagged-activated Notch signaling promotes angiogenesis [202] and stimulates bone marrow-derived stromal/stem cells (BMSCs) to promote skeletal regeneration [203]. Overall, these findings show the therapeutic potential of either restoring proper Notch signaling or stimulating *JAG1* overexpression as a mediator of the regenerative process in DMD muscles independent of the presence of dystrophin.

Increasing the number of asymmetric divisions can be achieved independent from the polarized dystrophin distribution at the membrane in activated SCs, e.g., via activation of epidermal growth factor receptor (EGFR) and Aurka signaling pathways [204]. Promising results were also achieved following *Wnt7a* overexpression in EDL muscle and Wnt7a protein injection into TA muscles of *mdx* mice. In addition to an increase in muscle strength and a shift of induction toward slow-twitch fibers, which reduced the level of contractile damage, this treatment also regenerated the SC pool via an enhancement of the symmetric division rate [205]. Presumably, noncanonical Wnt7a signaling stimulates SC symmetric expansion through the planar cell polarity pathway (polarizing distribution of VANGL2, a planar cell polarity effector) and myofiber hypertrophy through the AKT/mTOR  pathway [205, 206]. In turn, canonical Wnt signaling, which is elevated in *mdx* mouse muscles, was shown to activate the TGFβ pathway, resulting in a fibrogenic phenotype in SCs [207]. Importantly, suppression of factors upregulated in DMD that inhibit myogenesis, such as TGFβ, myostatin, and activin, is possible with follistatin treatment [112]. It is important to note, however, that in addition to its beneficial effects on SCs, follistatin can also have detrimental effects on tendons [111, 113].

RNA-seq profiling of SCs from *mdx* and control mice demonstrated altered expression of over 1000 genes in quiescent SCs and 3000 genes in activated SCs [194]. Interestingly, Boldrin et al. showed that *mdx* SCs retain their regenerative potential upon transfer to a healthy muscle environment [208], which shows that exploring SC signaling and SC interactions with surrounding tissues can reveal the potential for effective therapies.

## Cell signaling in the heart

Cardiac failure is the leading cause of premature mortality in DMD patients [209, 210]. Pathological symptoms develop with age and manifest in progressive fibrosis, left ventricular dilation, and overall reduced systolic function, resulting in cardiomyopathy and atrial arrhythmias [211]. Heart regeneration is particularly challenging because unlike skeletal muscle tissue, this organ lacks resident stem cells/satellite cells that could regenerate the cardiomyocyte population [212]. In healthy tissue, cardiomyocytes, fibroblasts, endothelial and vascular smooth muscle cells are in homeostatic equilibrium [213], whereas in dystrophic muscle, high levels of inflammation and fibrogenic cells in coronary advenitia aggravate the condition of the patients [214–216].

Cardiomyocyte death in DMD patients is associated with the absence of dystrophin-related mechanical functions and signaling. As in skeletal muscle, cardiomyocytes struggle with an overload of Ca^2+^, the absence of nNOS/NO signaling [217–219] and the overproduction of ROS, which lead to the progression of pathological alterations. Law et al. [220] summarized known mechanisms responsible for mishandling Ca^2+^ in dilating cardiomyopathy in DMD hearts and reported experimental therapeutic targets that could address this issue. In particular, excess Ca^2+^ and oxidative stress result in degeneration of mitochondria [221], which cannot be removed effectively in the process of mitophagy [222]. These changes are probably due to dysfunction in the Pink1/Parkin1 pathway, which regulates the process of cleaning cells from nonfunctional mitochondria [222]. Affected cardiomyocytes also release exosomes containing miRNAs, which have been found to aggravate DMD pathogenesis [223]. Furthermore, enhanced Tgfβ1 signaling in coronary adventitial cells was identified as a factor that induces fibrotic changes. In a study by Ieronimakis et al. overexpression of *Tgfβ1* ligand in endothelial cells stimulated coronary adventitial cells via the Tgfβ pathway to become fibrotic and produce type I collagen, resulting in perivascular fibrosis [215], which shows that targeting the TGFβ pathway in either skeletal or cardiac muscles could be a viable signaling-based therapeutic strategy in DMD. Therapeutic approaches to treat dystrophic cardiomyopathy that are currently in use or in development have been discussed in-depth in previous works [57, 224].

## Conclusions

Although dystrophin and utrophin genes are ubiquitously expressed in various tissues, significant full-length dystrophin synthesis is limited to a few cell types, including myofibers, cardiomyocytes, smooth muscle cells, neurons, endothelial cells, and satellite cells [25, 184, 225]. DMD patients suffer particularly as a consequence of the absence of full-length dystrophin in the striated muscle tissue and succumb to the disease after years of progressive and debilitating symptoms [4]. Some of the dystrophin-related roles pertain to its mechanical functions and widespread interactions with transmembrane as well as cytoskeletal proteins, such as ankyrins, microtubules, plectin,  γ-actin and cyotokeratins that enable efficient transmission of forces and structural plasticity [66, 226, 227]. The dystrophin-glycoprotein complex together with its associated proteins, however, is also responsible for the interaction with various signaling molecules [25, 184, 228] that regulate cell proliferation, migration or maintenance via, in some instances, epigenetic and transcriptional changes, such as in the process of myofiber repair [8, 9, 11]. Importantly, in addition to intracellular signaling, cell-to-cell communication is also affected in dystrophin deficiency, as shown, e.g., by vasoconstriction related to nNOS downregulation and displacement from the sarcolemma [121–124], abrogated satellite cell activation due to dysregulated Notch signaling [199], or changes in the production of myokines that affect the bone [71, 93, 98, 104]. Therapeutic approaches should thus focus not only on stopping myofiber degeneration and improving satellite cell division kinetics but also on normalizing abrogated signal transmission. For instance, although warranted by observations of relatively mildly affected Becker muscular dystrophy (BMD) patients, technological shortcomings and methodological feasibility, the rod domain of dystrophin has largely been ignored in either gene therapy- or antisense oligonucleotide-based therapeutic approaches [36]. However, the newly obtained data changed that paradigm and resulted in addition of the nNOS binding domain to rAAV vectors carrying laboratory designed dystrophin coding sequences [229]. The MARK2 binding site might also be included in constructs that could be used in satellite cell therapeutic strategies.

Sodium nitrate (NO donor) as well as inhibitors of myostatin, HDAC and connective tissue growth factor are currently in clinical trials [11, 230–235], with others, including a WNT7a-like compound, in preclinical development [236]. It is also important to note that corticosteroids significantly alleviate tissue damage by downregulating the activity of signaling molecules associated with chronic inflammation, and new treatments based on molecules with less severe side effects are being tested [237–239]. Therapeutic strategies modulating specific signaling pathways could be used either as adjuncts or stand-alone strategies. Importantly, approaches such as stimulation of EGFR-Aurka pathways or Jagged 1 upregulation have been successfully used in animal models and do not seem to require dystrophin to normalize the division kinetics of satellite cells or muscle function. Most of the treatments grounded on dystrophin/utrophin gene delivery or exon skipping aimed at restoring the reading frame do not provide the fully functional dystrophin protein, and signaling approaches might be required as complementary strategies. Disturbances in noncoding RNA levels in DMD also provide an alternative strategy to monitor the progression of the disease or treatment efficacy [67, 240], and noncoding RNAs are under research as potential therapeutic targets [241, 242]. Other approaches would also need to be used in severely affected patients who lack large amounts of functional tissue. Here, therapies based on the reversal of pathological symptoms, such as conversion of fibroblasts to myoblasts by the induction of MYOD, could be employed [243].

In summary, DMD should be understood as a disease of not only affected myofibers and satellite cells but also a disorder in which abrogated communication between different cell types occurs. Therefore, targeting affected signaling pathways in patients is a promising treatment strategy for DMD. We believe that by taking this systemic view, we can achieve safe and holistic treatment that can restore correct signal transmission and gene expression in diseased DMD tissues.
